# A novel approach for the synthesis of nanostructured Ag_3_PO_4_ from phosphate rock: high catalytic and antibacterial activities

**DOI:** 10.1186/s13065-021-00767-w

**Published:** 2021-06-30

**Authors:** Karim Dânoun, Rida Tabit, Abdelaziz Laghzizil, Mohamed Zahouily

**Affiliations:** 1 MASCIR Foundation, VARENA Center, Rabat Design, Rue Mohamed El Jazouli, Madinat AlIfran, 10100 Rabat, Morocco; 2 Laboratory of Materials, Catalysis & Valorization of Natural Resources, URAC 24, Faculty of Sciences and Technology, Hassan II University of Casablanca, B.P. 146, 20650 Casablanca, Morocco; 3 Laboratory of Applied Chemistry of Materials, Faculty of Science, Mohammed V University, Rabat, Morocco

**Keywords:** Nanostructured Ag_3_PO_4_, Natural phosphate, Nitrophenol reduction, Antibacterial studies

## Abstract

**Background:**

Silver orthophosphate (Ag_3_PO_4_) has received enormous attention over the past few years for its higher visible light photocatalytic performance as well as for various organic pollutants degradation in aqueous media. Therefore, considerable efforts have been made to the synthesis of Ag_3_PO_4_ with high catalytic efficiency, long lifetime, and using low-cost inorganic precursors.

**Results:**

This article describes our efforts to develop a novel approach to synthesize of nanostructured silver phosphate (Ag_3_PO_4_) using phosphate rock as alternative and natural source of PO_4_^3−^ precursor ions. The catalytic experimental studies showed that the nanostructured Ag_3_PO_4_ exhibited excellent catalytic activity for reduction of p-nitrophenol in the presence of NaBH_4_ at room temperature. Furthermore, the antibacterial studies revealed that the obtained Ag_3_PO_4_ possess significant effect against E. *Coli* and S. *Aureus* bacteria.

**Conclusion:**

The obtained results make the nanostructured Ag_3_PO_4_ prepared from natural phosphate as a highly promising candidate to be used as efficient catalyst and antibacterial agent.

**Graphic Abstract:**

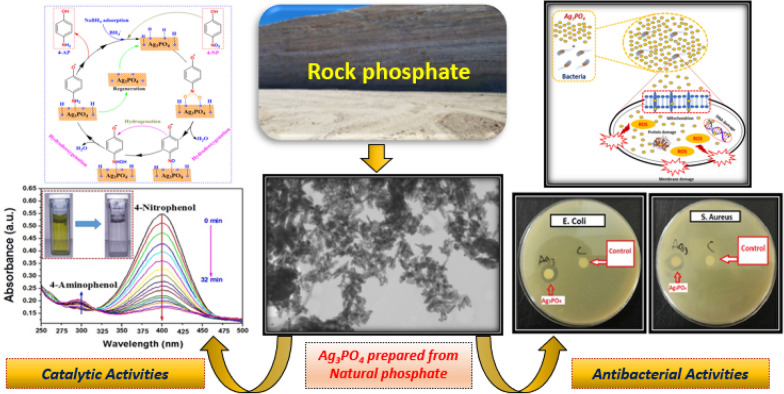

## Introduction

With industrialization processes and more human activities, the environmental contamination caused by organic pollutants is becoming an overwhelming mystery all over the world [1, 2]. The phenolic compounds are one of the most notorious pollutants generated by industrials sources such as synthetic intermediate in the manufacture of pharmaceuticals, plastics, pigments, dyes, pesticides and fungicidal agents, explosives and industrial solvents [3]. Due to their potential to harm human health and living organisms at low concentrations, these compounds were classified as priority materials by the United States Environmental Protection Agency (USEPA) among the top 114 organic pollutants [4, 5]. Among the different phenolic compounds, 4-nitrophenol (4-NP) is one of the most frequently occurring products [6]. However, the 4-aminophenol (4-AP) is one of the most important intermediates in the preparing of several analgesic and antipyretic drugs like paracetamol, acetanilide, and phenacetin [7]. Also, it is utilized as corrosion inhibitor in paints, and anticorrosion-lubricating agent in fuels. Further, 4-aminophenol is used efficiently in the dye industry as a wood stain and a dyeing agent for fur and feathers [8]. In view of the harmful effect of 4-NP and the growing demand for 4-AP, the conversion of 4-NP directly to 4-AP via catalytic route becomes greatly desirable. Various metal nanoparticles like as platinum, gold, copper, ruthenium and palladium, are used intensively for the reduction of nitrophenols compounds. Although, all these catalysts are very expensive for industrial use in bulk quantity. Consequently, considerable effort has been devoted to exploit new and efficient photocatalysts for environmental pollution control. Of the well-known photocatalyst materials, silver orthophosphate (Ag_3_PO_4_) has attracted great attention over the past few years due to its excellent photocatalyst ability for water splitting and degradation of organic contaminants [9–14] Several methods have been reported in the literature for the synthesis of Ag_3_PO_4_ with variety of commercial reagents to improve of its photocatalytic properties, such as rare metal doping, surface modification, semiconductor coupling and formation of noble metal composites [15–18]. Nevertheless, some of these synthetic routes suffer from several drawbacks since they involve long and complex steps to produce the desired product. Moreover, many chemical reagents seem to be necessary making the process more costly. However, low-cost fabrication of well-defined Ag_3_PO_4_ with superior catalytic properties via a simple process remains a great challenge. On the other hand, natural phosphates are an important natural resource in Morocco, which needs to be valorised. They can be employed not only as fertilizers but also, they have been exploited effectively as catalysts in a wide range of organic transformation [19–21]. In continuation of our ongoing program to develop an interesting catalyst at low-cost [22–27], we describe in this paper, a novel chemical wet method-based dissolution–precipitation reactions using Moroccan natural phosphate (NP) as phosphorus precursor to synthesize of the nanostructured Ag_3_PO_4_ as catalyst for the reduction of 4-NP to 4-AP in the presence of sodium borohydride using aqueous phase and its antimicrobial activity against *Escherichia coli* and *Staphylococcus aureus* bacteria. To the best of our knowledge, no studies have been performed on the development of Ag_3_PO_4_ from natural phosphate and testing its catalytic activity for the reduction reaction in aqueous solution, and its antibacterial activity.

## Experimental section

### Materials

Silver nitrate AgNO_3_ (≥ 99.0%), Ammonia hydroxide solution NH_4_OH (28%), 4-nitrophenol (C_6_H_5_NO_3_), and Sodium borohydride (NaBH_4_) were purchased from Sigma-Aldrich. All reagents were of analytical grade and used as received. Deionized water was utilized through all the preparation procedures.

### The preparation of nanostructured Ag_3_PO_4_ catalyst

**Scheme 1:**
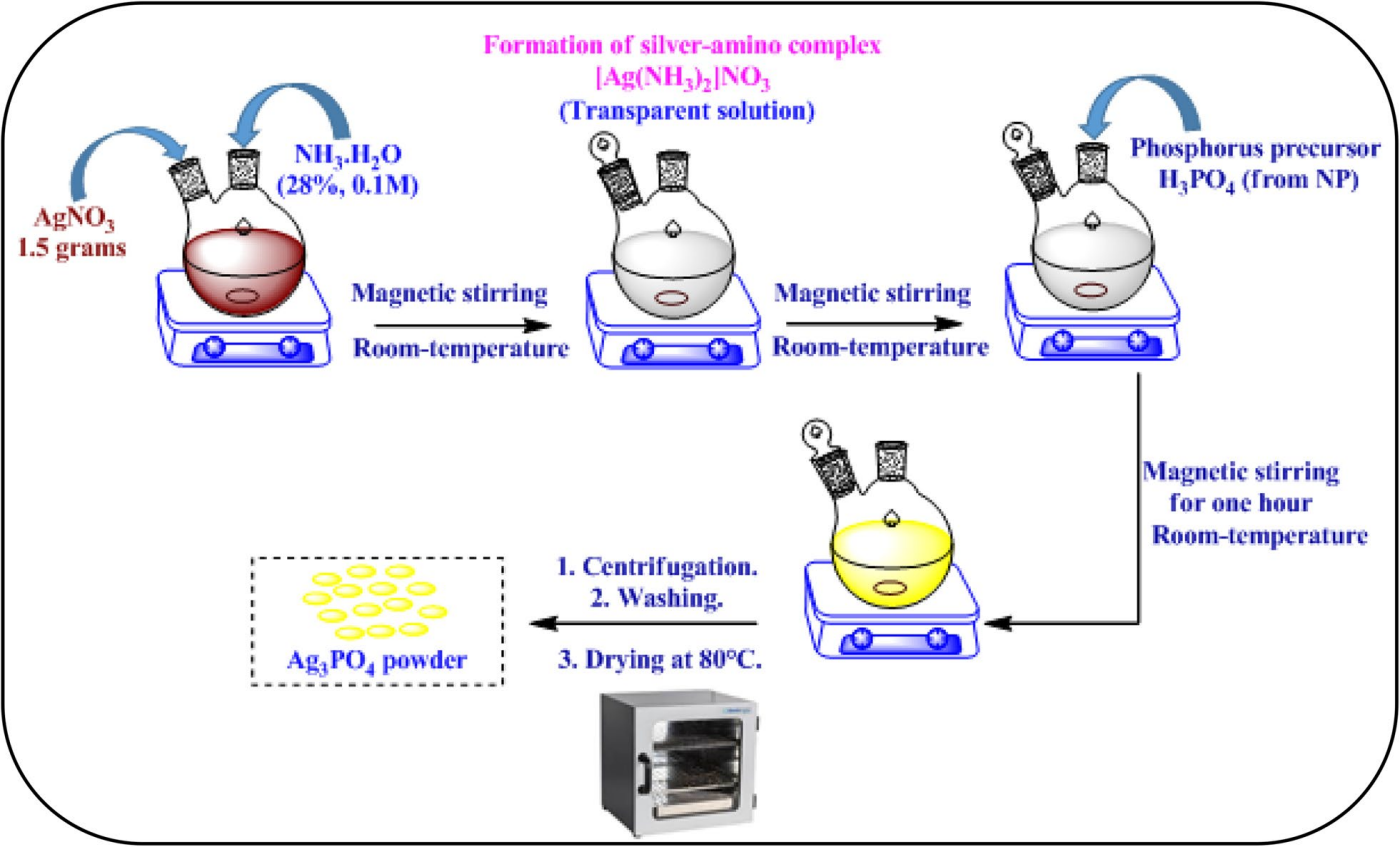
Schematic diagram of the preparation of nanostructured Ag_3_PO_4_ by dissolution–precipitation process from natural phosphate

The Ag_3_PO_4_ was prepared by a dissolution/precipitation method from a natural phosphate rock coming from the Khouribga region (Morocco). To use this material prior requires initial treatments such as crushing and washing. The fraction of 200–400 μm grain size was washed with distilled water several times to remove the soluble matter. The different elemental constituents of this mineral are given in Table 1. Then, the dissolution process was carried out in a round bottom flask of 500 ml capacity at a rate of 200 rpm. Firstly, about thirty grams of natural phosphate was dissolved in deionized water acidified by HNO_3_ acid (65%) to pH 2, under continuous stirring at room temperature. We obtained H_3_PO_4_ and Ca^2+^ ions as well as the insoluble matter after solid/liquid separation process by centrifugation. Next, the Ag_3_PO_4_ catalyst was prepared by a simple precipitation method. In a typical synthesis, 1.5 g of AgNO_3_ (8.83 mmol) was dissolved in 80 ml deionized water over 10 min, then the ammonia hydroxide solution 28% (0.1 M) was added with drop by drop under magnetic stirring to above mixture to form a transparent solution. And then, phosphorus precursor prepared previously from natural phosphate were added gradually to the mixture reaction, the resulting precipitate was magnetically stirred at room temperature for one hour. After that, a yellow precipitate of silver phosphate Ag_3_PO_4_ is then obtained by centrifugation, which is then washed several times with deionized water to release any unreacted species such as Ca^2+^ and NO^3−^ ions. Finally, the obtained powder Ag_3_PO_4_ was dried in desiccator at 80 °C overnight. The schematic illustration of preparation process of nanostructured Ag_3_PO_4_ from natural phosphate is exhibited step by step in Scheme 1.

**Table 1:** Khouribga natural phosphate analysis

Elements	P_2_O_5_	CaO	CO_2_	SiO_2_	F	Al_2_O_3_	SO_3_	Fe_2_O_3_
(%)	30.74	50.47	6.64	5.91	3.60	0.43	1.83	0.2

### Characterization of the catalyst

X-ray diffraction (XRD) patterns were obtained at room temperature on a Bruker AXS D-8 diffractometer using Cu-Kα radiation in Bragg–Brentano geometry (θ-2θ). Fourier transform infrared (FT-IR) was performed on an ABB Bomem FTLA 2000 spectrometer equipped with a Golden Gate single reflection ATR accessory. The Raman spectra were recorded in the range from 400 to 1400 cm^−1^ with a Thermo Scientific DXR2 spectrometer. Scanning electron microscopy images were recorded on a FEI Quanta 200 microscope after carbon metallization. Specific surface area was determined from the nitrogen adsorption/desorption isotherms (at-196 °C) and measured with a Quantachrome Autosorb-1 automatic analyzer using the BET equation.

### Catalytic reduction of 4-nitrophenol to 4-aminophenol

The catalytic activities were evaluated by reduction of 4-nitrophenol (4-NP) to 4-aminophenol (4-AP) in a quartz cuvette. In a typical procedure, 100 µL of 4-NP (1 mM) and 1 mL NaBH_4_ (0.1 M) were placed in a quartz cuvette containing 3 mL of deionised water. After that 5 mg of Ag_3_PO_4_ was added into the cuvette to start the reduction reaction. The process of the conversion of 4-NP to 4-AP was followed by UV–Vis spectroscopy at a maximum wavelength of 400 nm.

### Antibacterial activity of nanostructured Ag_3_PO_4_ powder

The antibacterial activity of the nanostructured Ag_3_PO_4_ was studied on *Staphylococcus aureus* (S. aureus) and *Escherichia coli* (E. coli) by the standard disk diffusion assay on Muller-Hinton agar medium. All disks and materials were sterilized in an autoclave at 120 °C for 20 min before experiments. The disk diffusion assay was performed by placing a 6 mm disk saturated with 10 µL of Ag_3_PO_4_ aqueous dispersions (1000–125 µg/mL) onto an agar plate seeded with *E. coli* or *S. aureus*. After 24 h of incubation at 37 °C, the diameters of the inhibition zones were measured.

## Results and discussion

### Characterization of nanostructured Ag_3_PO_4_

**Fig. 1:**
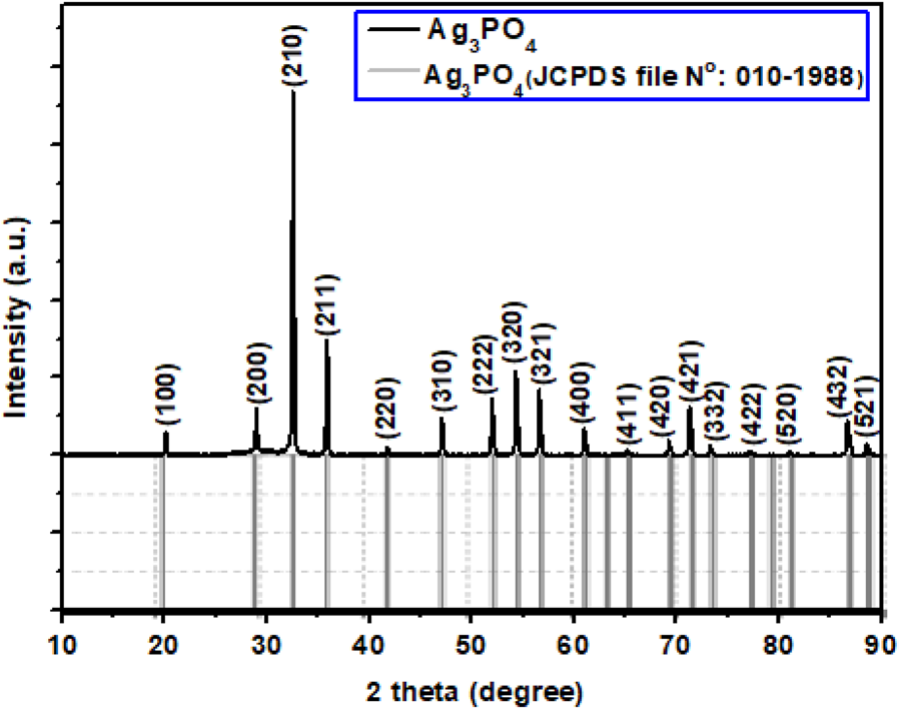
X-ray diffraction pattern of nanostructured Ag_3_PO_4_

The phase structures of the as-prepared nanostructured Ag_3_PO_4_ were investigated by XRD and showed in Fig. 1.

**Fig. 2:**
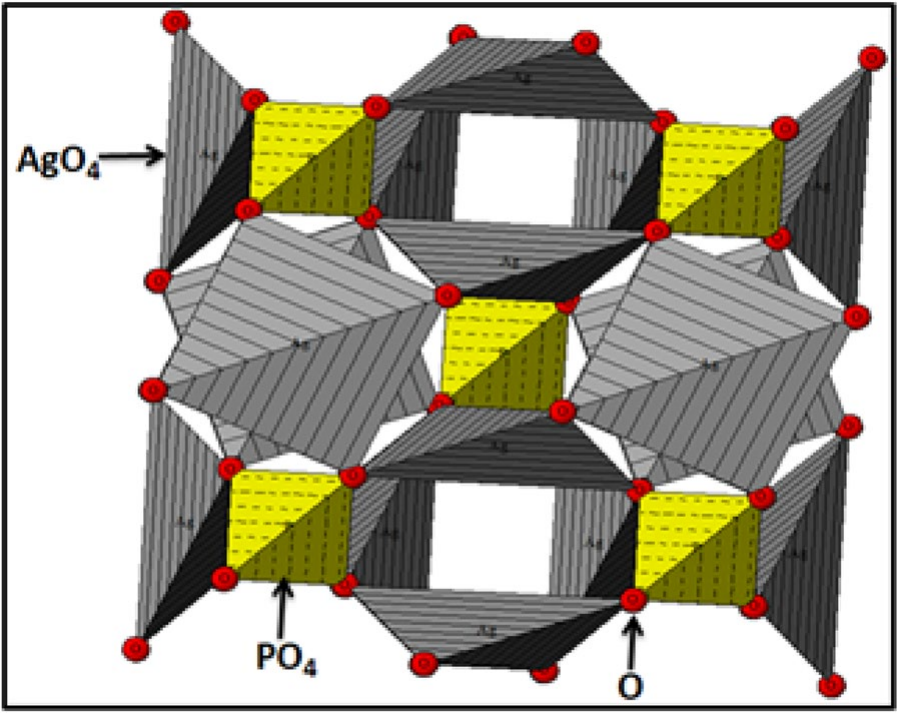
Projection view of nanostructured Ag_3_PO_4_along [100] direction

X-ray diffraction pattern of nanostructured Ag_3_PO_4_ clearly showed that all diffraction peaks are a near-systematic superposition of those of standard structure of Ag_3_PO_4_ (JCPDS file N^o^010-1988). No diffraction peaks for other phases such as calcium phosphate are detected, this indicates that Ag^+^ cations have the best chemical affinity towards PO_4_^3−^ anions compared to that of Ca^2+^ ions originating from natural phosphate in which they have been cleaned from the solid by washing treatment. The obtained Ag_3_PO_4_ crystalizes in the cubic system with the space group P-43n (No.218) with a unit cell length of 6.002 Å. According to the Debye–Scherrer equation, the average crystallite size of the synthesized Ag_3_PO_4_ is about 39 nm. Furthermore, the atomic positions and the lattice parameters collected from Rietveld refinements were used as input data in structural analysis software to model the cubic Ag_3_PO_4_ structure as shown in Fig. 2. From the modeled structure, both Ag and P atoms are coordinated to four oxygen (O) atoms, resulting in tetrahedral [AgO_4_] and [PO_4_] clusters. Each isolated [PO_4_] cluster is bonded to three neighboring [AgO_4_] clusters by means of O atoms. According to the qualitative results, the average of P-O and Ag–O distance is about 2.37 Å and 1.54 Å, respectively.

**Fig. 3:**
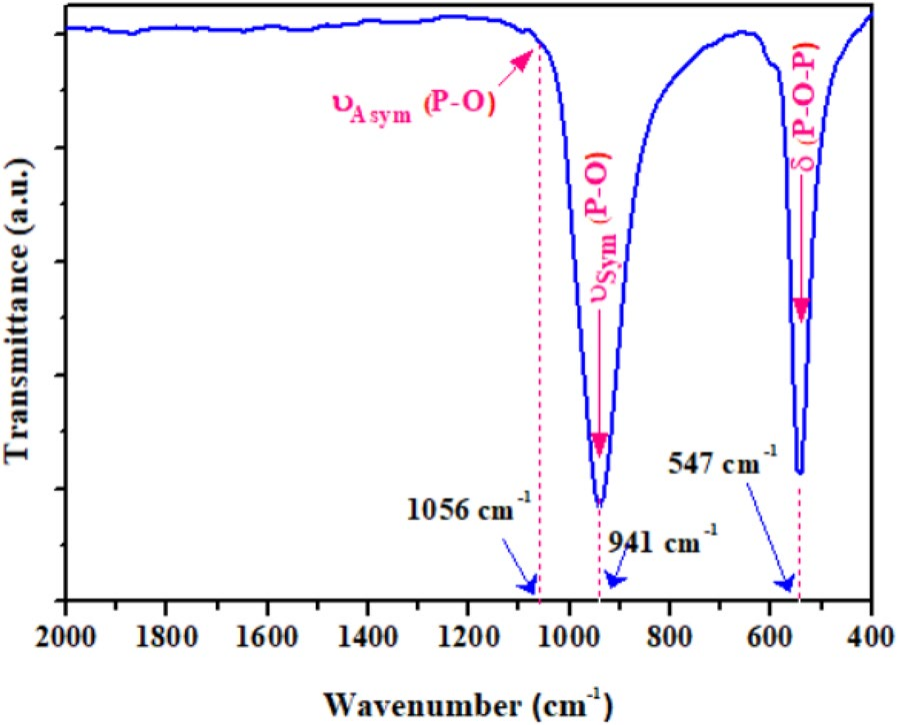
FT-IR spectra of nanostructured Ag_3_PO_4_ powder

Figure 3 showed the Fourier transform infrared (FT-IR) spectrum of the Ag_3_PO_4_ sample, exhibiting two strong bands in the 1072–732 cm^−1^ and 586–447 cm^−1^ ranges related to the molecular vibrations of PO_4_ groups in Ag_3_PO_4_ sample. The former band centered at 941 cm^−1^ with a very small shoulder at around 1056 cm^−1^ is assigned to the symmetric and asymmetric stretching mode of the P–O bonds, while the latter cantered at 547 cm^−1^ is related to deformation from the bending mode of the P–O–P bonds.

**Fig. 4:**
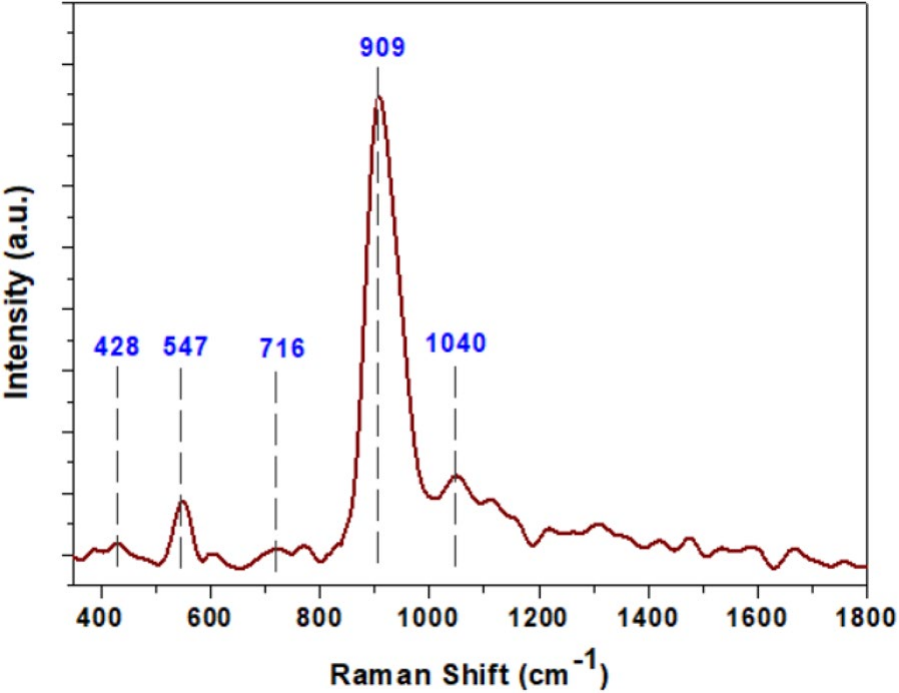
Raman spectrum of nanostructured Ag_3_PO_4_

Furthermore, the Raman spectroscopy was also employed to confirm the FTIR obtained resultants and phase purity of the as-prepared powder. As shown in Fig. 4, the band located at 909 cm^−1^ is attributed to symmetric stretching vibrations of [PO_4_] clusters, while the asymmetric stretching vibrations of this cluster were verified at 1040 cm^−1^. The bending vibration modes related to [PO_4_] clusters were found at 428 and 547 cm^−1^. The band centered at 716 cm^−1^ is ascribed to symmetric stretching vibrations of P-O-P bonds. These obtained results are in good agreement with those reported in the literature [28].

**Fig. 5:**
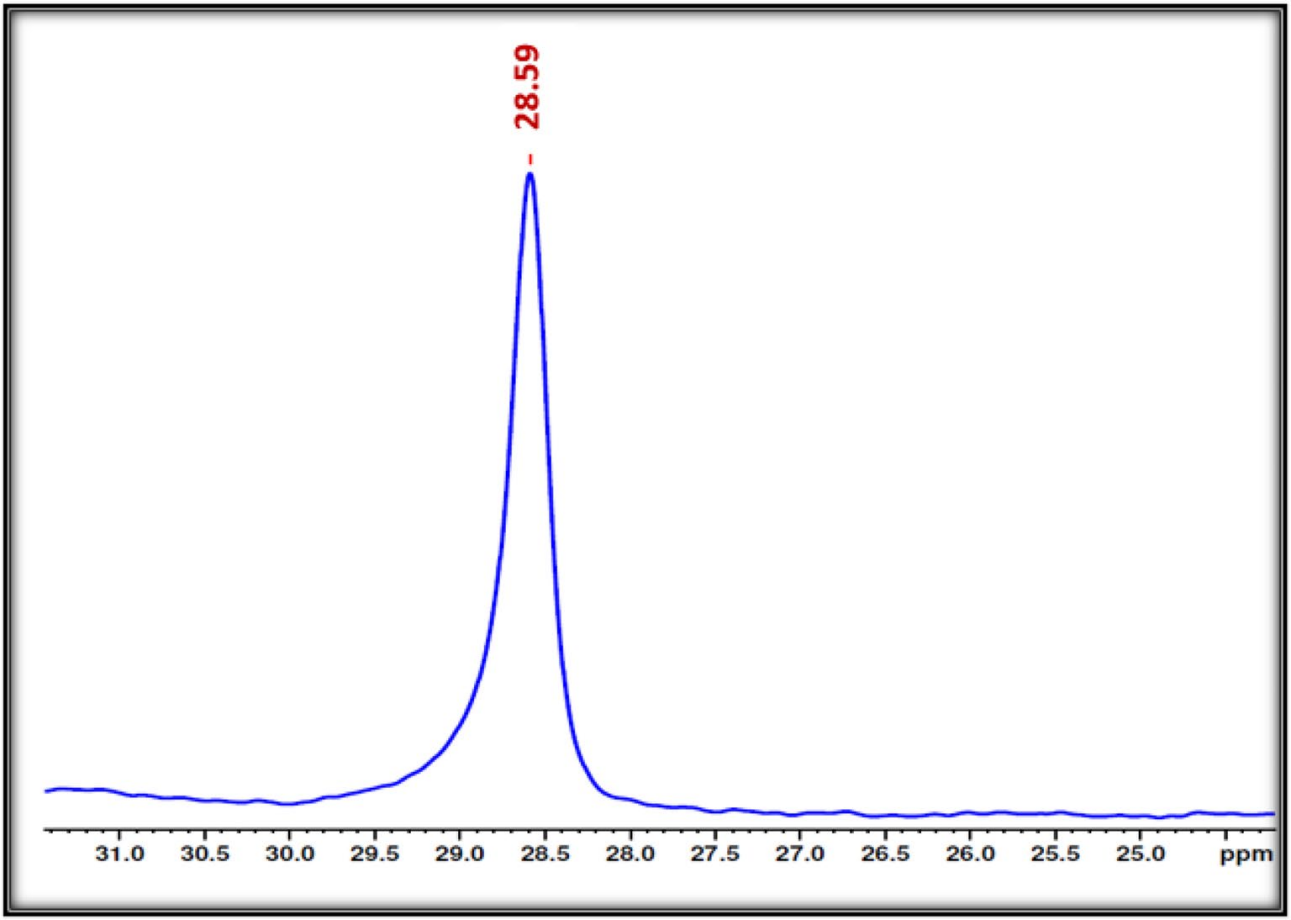
Solid-state ^31^P-MAS NMR spectrum of nanostructured Ag_3_PO_4_ prepared from natural phosphate rock

In order to support the afore mentioned interpretation ^31^PMAS-NMR studies were also investigated. As shown in Fig. 5, the presence of one single crystallographic site of phosphorus at a chemical shift of δ = 28.59 ppm, proves the existence of only one type of phosphorus site in the Ag_3_PO_4_ material. Thus, these results are in good accordance with the previously data obtained by XRD analysis and confirm the presence of pure phase of obtained nanostructured silver phosphate [29].

**Fig. 6:**
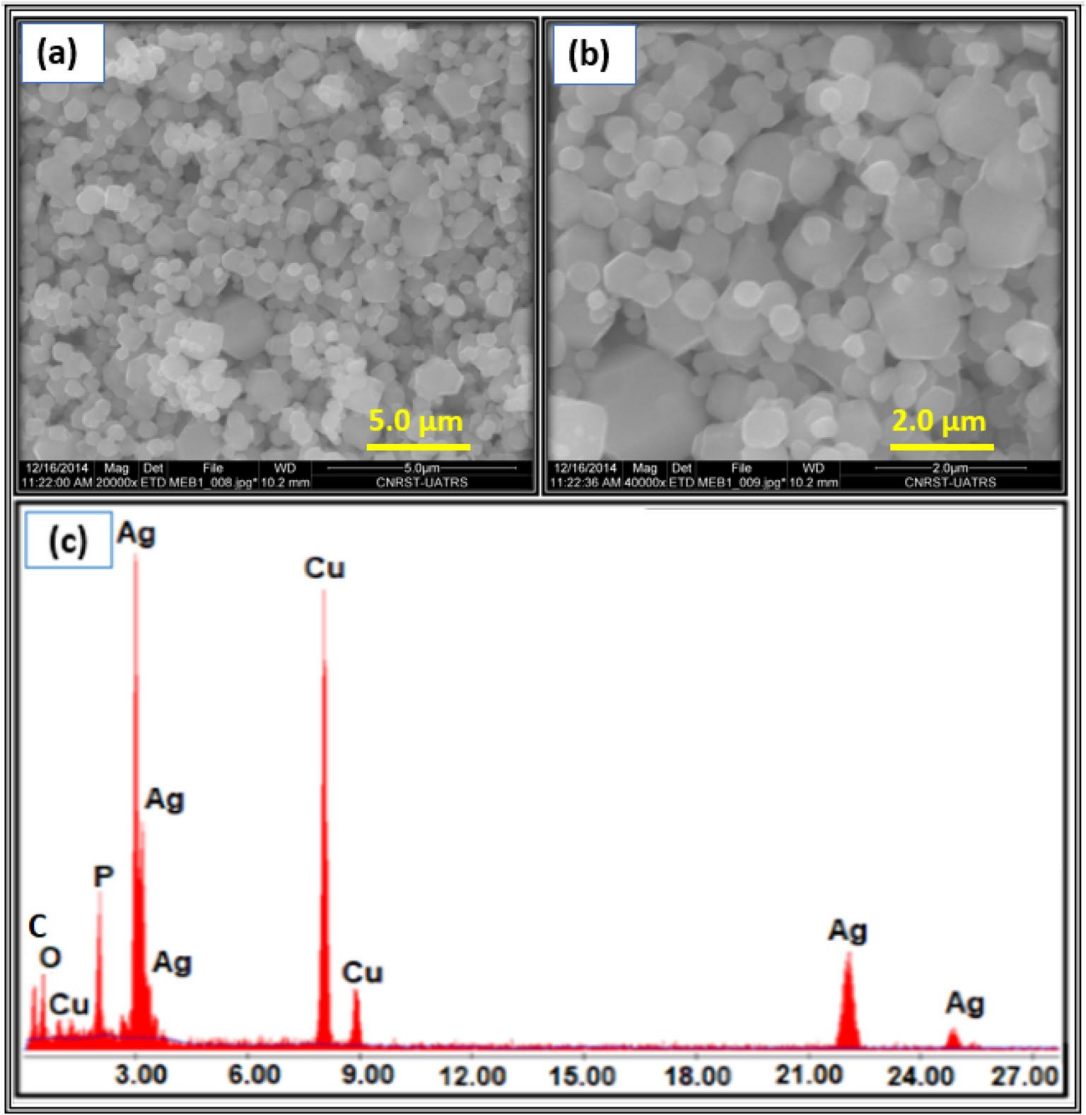
**a**, **b** SEM images and **c** EDS spectrum of the prepared Ag_3_PO_4_ powder

To better elucidate the morphological properties of the prepared Ag_3_PO_4_ from natural phosphate, SEM analysis was carried out. In the lower magnification images, the Fig. 6a indicates that the surface of nanostructured Ag_3_PO_4_ is formed by a large amount of quasi-spheroid particles having hexagonal and cubic structures, while the higher magnification image, as shown in Fig. 6b, clearly reveals the quasi-spheroid particles through non-uniform diameter polyhedrons. On the other hand, to confirm the chemical composition of the nanostructured Ag_3_PO4, a semi-quantitative elemental analysis was performed, and its EDS spectrum showed in Fig. 6c. The obtained results revealed the presence of O, P and Ag elements without any calcium traces indicating that the Ag_3_PO_4_ prepared is pure and does not contain any impurities. Note that the presence of carbon and copper peaks is originated from adhesive Cu-carbon tape.

**Fig. 7:**
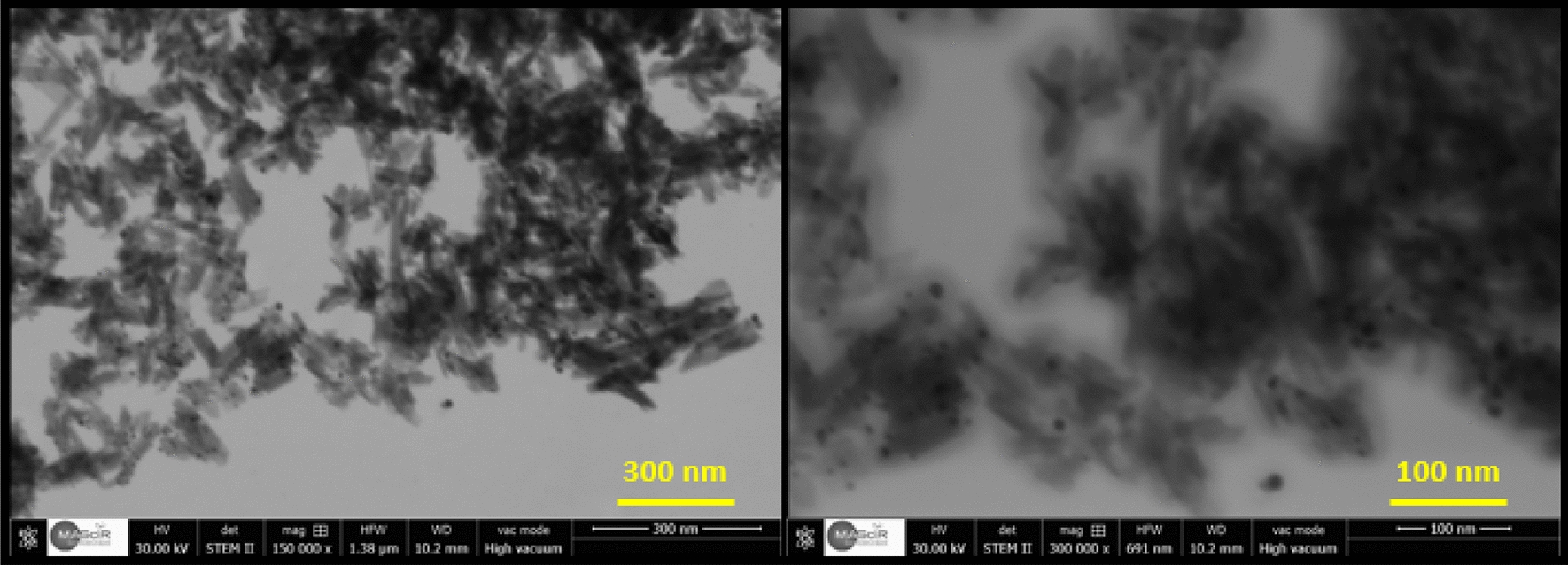
STEM micrographs of nanostructured Ag_3_PO_4_ prepared from naturel phosphate rock

In order to provide more details about the morphological properties regarding the obtained nanostructured Ag_3_PO_4_, the scanning-transmission electron microscope (STEM) was also used (Fig. 7). The STEM data acquired showed that the Ag_3_PO_4_ particles were clustered and formed heterogeneous aggregates of particles that were different in size and irregularly formed.

**Fig. 8:**
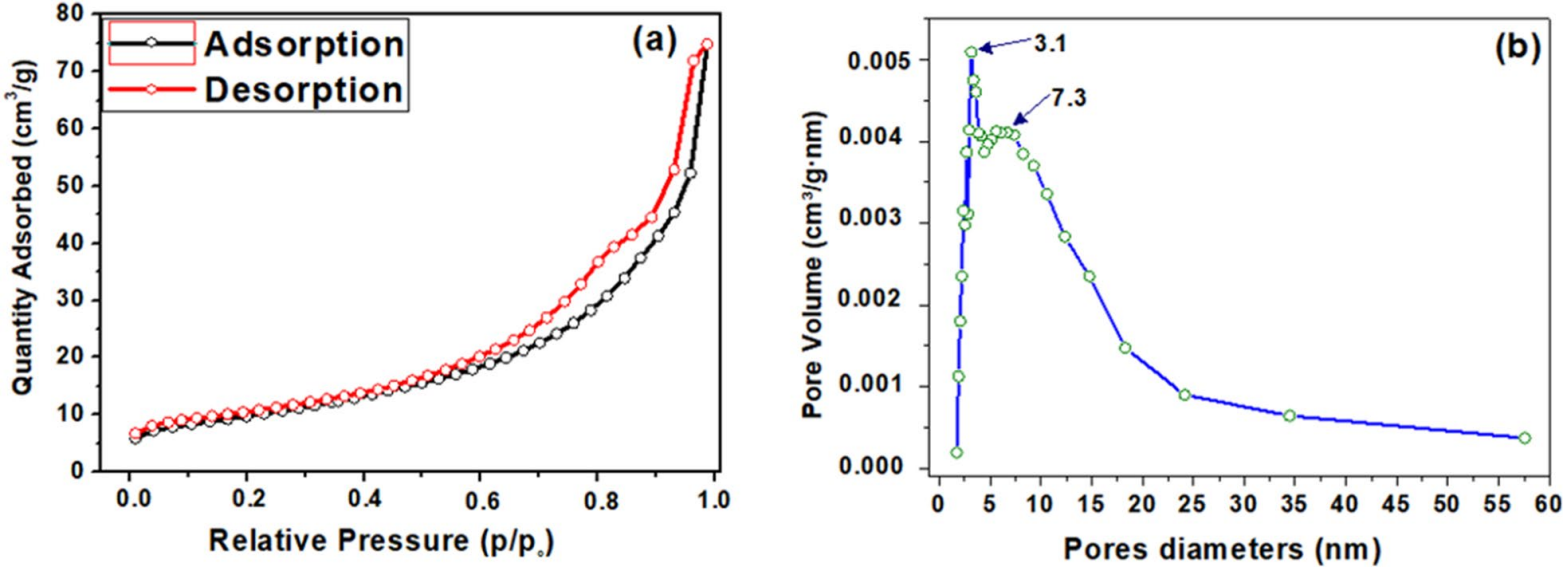
**a** N_2_-sorption isotherms and **b** BJH pore size distribution of Ag_3_PO_4_ prepared form NP

To further demonstrate the porous structure of nanostructured Ag_3_PO_4_, the specific surface area (S_BET_) of the Ag_3_PO_4_ powder was calculated from N_2_-sorption measurements and application of the BET method. As shown in Fig. 8a, the sorption isotherm exhibited a type IV isotherm according to the IUPAC classification with a distinct hysteresis loop of H3. Its specific surface area was of 35 m^2^/g and average pore size *D*_*p*_ calculated from BJH (Barrett-Joyner-Halenda) method was 3.1 nm and 7.3 nm (Fig. 8b). Comparing with the low values given in the literature, a relatively large porous surface of Ag_3_PO_4_ catalyst could provide more active and beneficial sites for the adsorption of target molecules through the active sites of the catalyst, which would promote the catalytic reaction.

### Catalytic Reduction of 4-NP to 4-AP

**Scheme 2:**
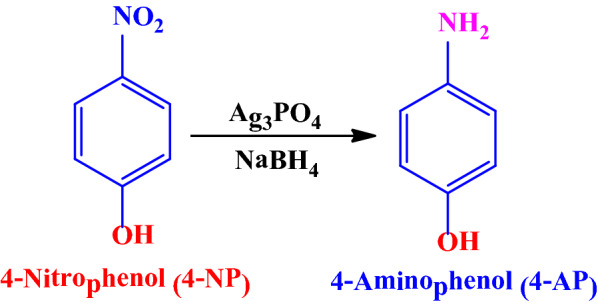
Representation of the overall reaction process for converting 4-NP to 4-AP by NaBH_4_ in the presence of nanostructured Ag_3_PO_4_ powder

To investigate the catalytic activity of the Ag_3_PO_4_ as nanostructured catalyst, the reduction of 4-nitrophenol to its corresponding amino derivatives, 4-aminophenol, in the presence of NaBH_4_ in aqueous media was chosen as a model reaction (Scheme 2). Currently, the reduction of 4-NP to 4-AP is monitored by UV–vis spectra at their specific wavelengths 317 nm for 4-NP and 300 nm for 4-AP.

**Fig. 9:**
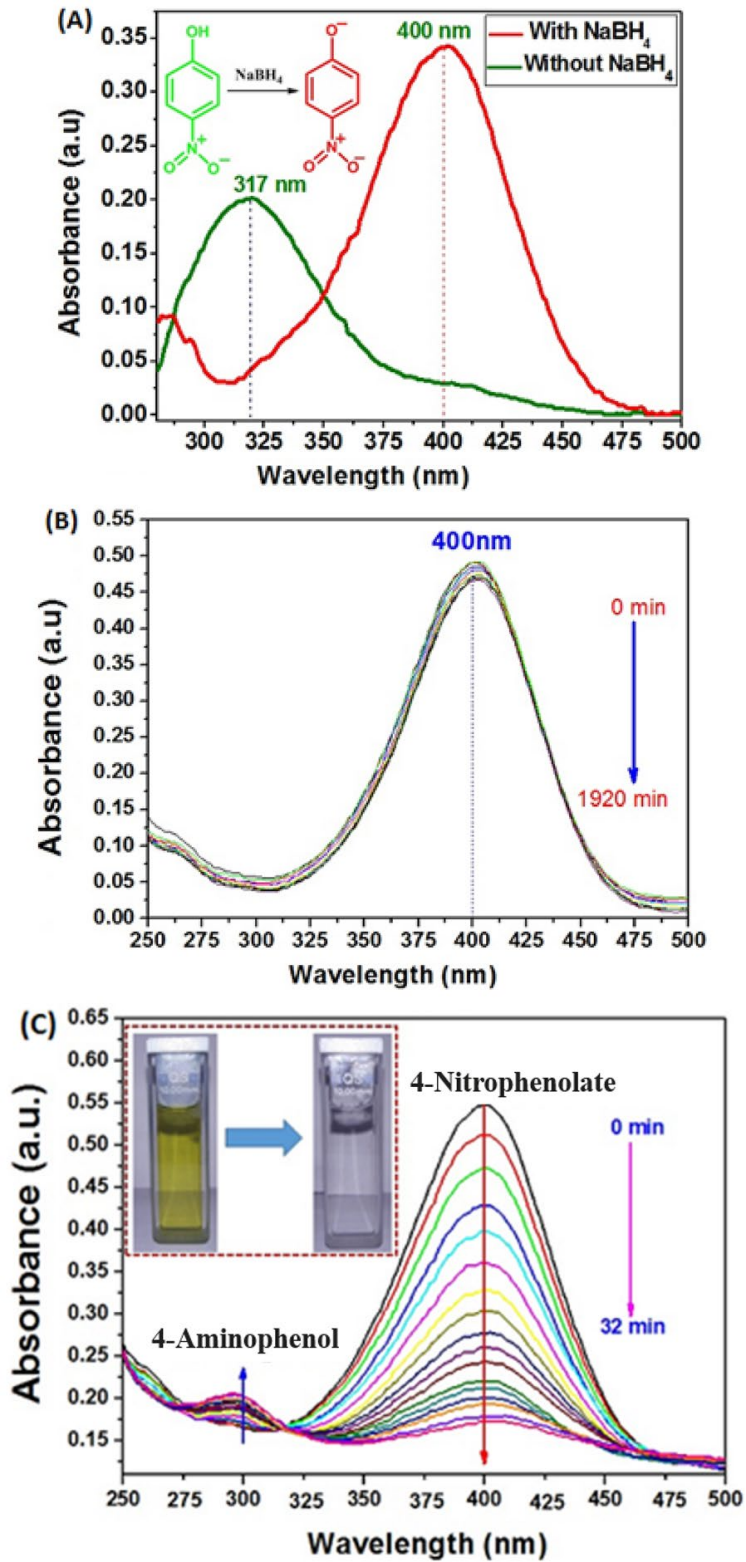
**a** UV–vis spectra of aqueous solutions of 4-NP with and without NaBH_4_, **b** UV–vis spectral evolution of solutions of 4-NP in the presence of NaBH_4_ without catalyst, **c** in the presence of nanostructured Ag_3_PO_4_ catalyst

Firstly, the ability of NaBH_4_ to reduce 4-NP in absence of our catalyst was examined. As shown in Fig. 9A, the 4-NP in an aqueous solution has a maximum absorption at 317. After added NaBH_4_ into solution, the absorbance peak of 4-NP was red shifted from 317 to 400 nm immediately along with a colour change from light yellow to bright yellow. This peak was due to the formation of 4-nitrophenolate ions in alkaline condition caused by the addition of reducing agent, as supported elsewhere [30]. However, in the absence of our catalyst the thermodynamically favorable reduction of 4-nitrophenol was not watched and the absorbance peak corresponding to 4-nitrophenolate ions at 400 nm rest unchanged for a long time (Fig. 9B). Then, when a small amount of Ag_3_PO_4_ nanostructured (5 mg) was introduced into reaction solution, the absorbance peak at 400 nm decreases significantly within 32 min and concomitant appearance of a new peak at 300 nm. The new absorption at 300 nm is characteristic peak of 4-AP, revealing the reduction of 4-NP to form 4-AP. In addition, as seen in the UV–Vis spectra (Fig. 9C), the presence of an isobestic point at 317 nm indicating that the catalytic reduction of 4-nitrophenol gives 4-aminophenol only without by product [31, 32].

**Fig. 10:**
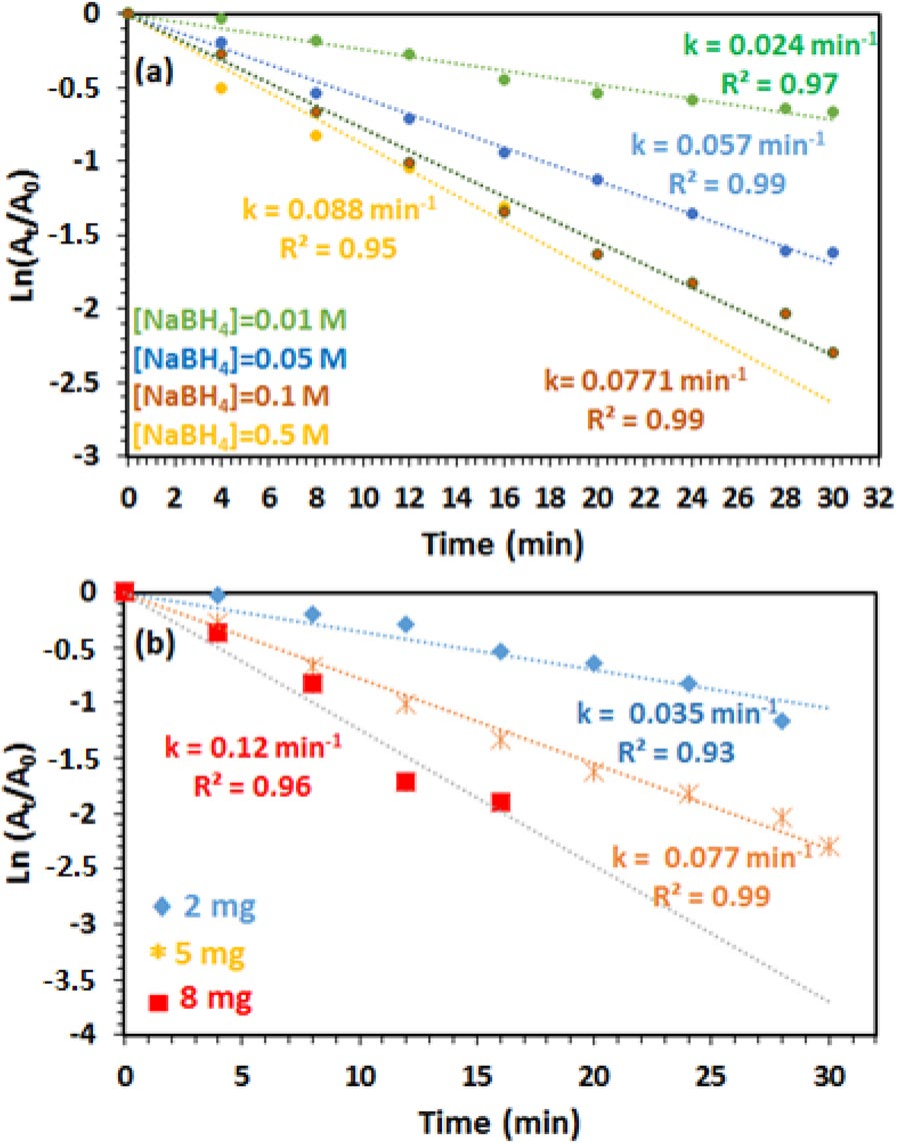
Logarithm of the absorbance at 400 nm versus reduction time of 4-NP under different conditions: **a** [NaBH_4_] molar concentration **b**, and amount of catalyst

To understand the catalytic conversion kinetic of 4-NP, the pseudo-first-order model was used: ln(*C*_t_/*C*_*0*_) = ln(A_*t*_/A_*0*_) = -*k*_*app*_* t*, where *C*_0_ and *C*_*t*_ are the 4-NP concentrations at t = 0 and t = t, respectively and *k*_*app*_ is the apparent rate constant, which is in good relationship with the disappearance of the nitrophenonlate band at 400 nm versus time. In this light, the influence of the NaBH_4_ molar concentration on the catalytic efficiency of the reduction of 4-NP into 4-AP was studied. Figure 10a shows the ln(A_t_/A_0_) plot as a function of time at different concentration of reducing agent (0.01; 0.05; 0.1 and 0.5 M) at room temperature. Based on the results of this study, it is clearly seen that the constant rate conversion of 4-NP to 4-AP increases from 0.024 min^−1^ to 0.088 min^−1^ with increasing NaBH_4_ concentration from 0.01 to 0.5 M, respectively. These results can be interpreted by the presence of an excess of the reducing agent, which favouring the diffusion of BH_4_^−^ ions on the catalyst surface by accelerating the reduction of the diffused 4-NP [33, 34]. Note that no significant change in apparent rate constant above 0.1 M of [NaBH_4_] was observed; thus, an optimum concentration of 0.1 M [NaBH_4_] was chosen for the future experiments. In addition, the effect of amount of the Ag_3_PO_4_ nanostructured on catalytic efficiency was also studied using 0.1 M of NaBH_4_ at room temperature. We should mentioned that the reaction was started after adding of Ag_3_PO_4_ as a nanostructured catalyst and the colour of the solution changed gradually from bright yellow to colourless indicated the successive reduction of 4-NP. As showed in Fig. 10b, the conversion reaction seems to be sensitive to the catalyst amount, but from 5 mg of the catalyst, the reaction became uncontrollable and ends very quickly. Thus, the optimum amount of the catalyst was selected to be 5 mg.

**Scheme 3:**
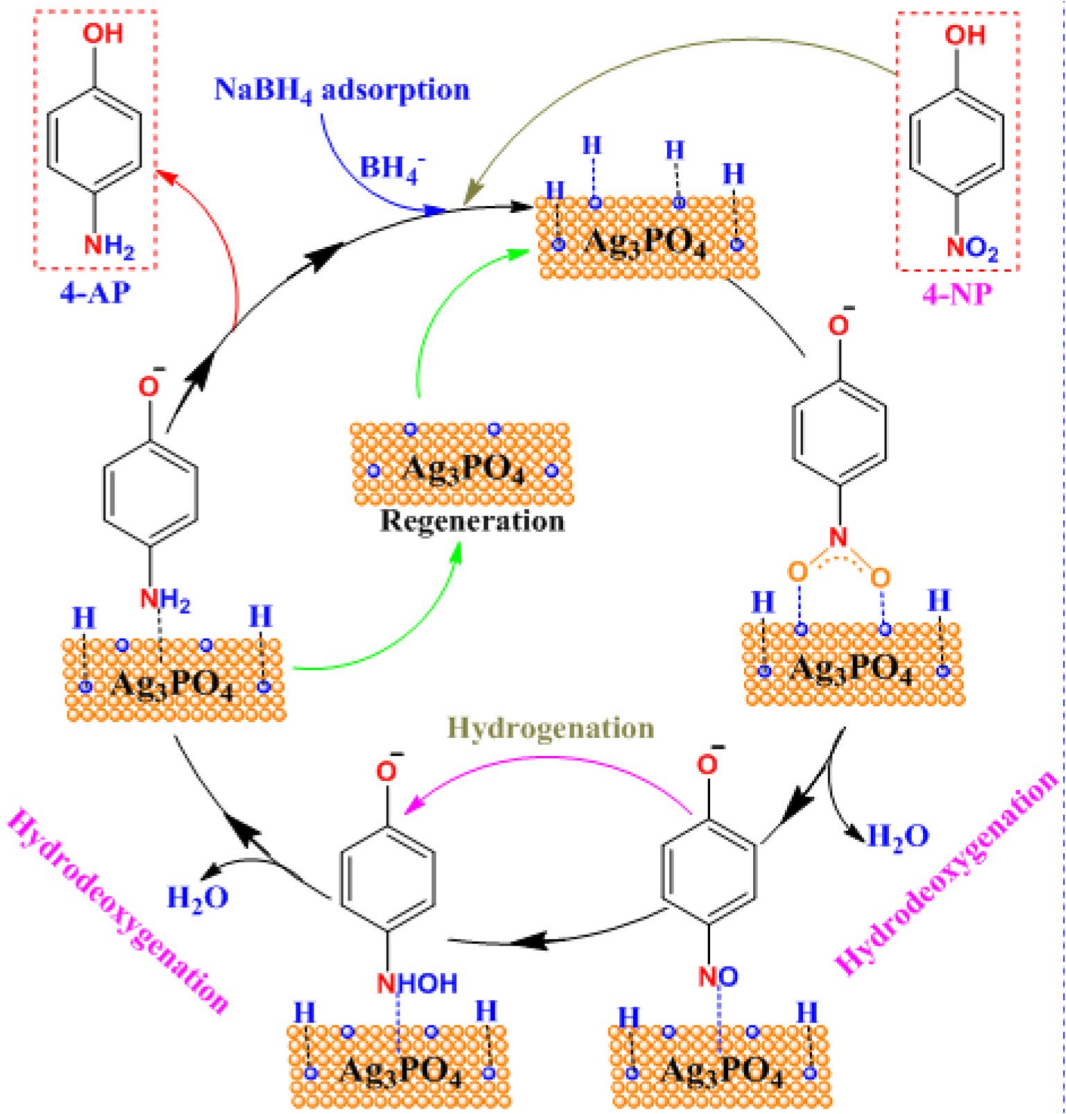
Mechanism for the reduction of 4-NP by the NaBH4 with nanostructured Ag3PO4 catalyst

Based on the results described above the proposed mechanism for the reduction of 4-NP is given in schematic (Scheme 3). As published elsewhere [35–37], the hydrogen atom of BH_4_^−^ is positively charged and could create fine electrostatic attractions with negatively charged oxygen from nitro groups at catalyst surface, facilitating the removal of oxygen and reduction of nitro groups. In addition, the residual nitrogen of -NO_2_ is also negatively charged due to its greater electronegativity than carbon from the benzene ring, and the H atoms of the positively charged H_2_O molecules could easily combine with the residual nitrogen of the nitrophenol to form the final aminophenol product. Adding to the proton transfer and deoxygenation, electron transport must occur simultaneously from the BH_4_^−^ clusters to 4-NP via the Ag_3_PO_4_ catalyst substrate to compensate for the charge balance and accomplish the process of reduction.

**Fig. 11:**
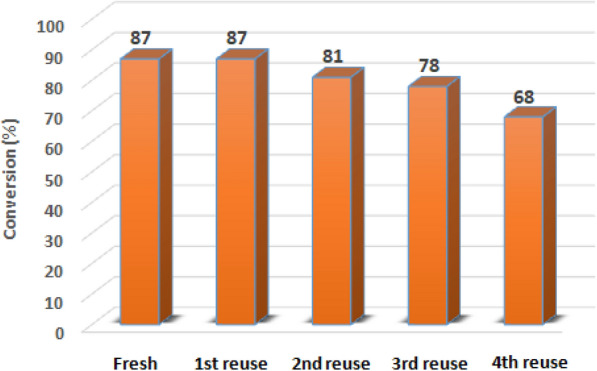
Reuse performance of nanostructured Ag_3_PO_4_ catalyst in reduction of 4-nitrophenol to 4-aminophenol

The reusability of the catalyst is another important factor from economic and environmental point of view, which it is highly desirable to examine in this study. At the end of the reaction, the catalyst was easily separated by filtration from solution, washed with deionized water and ethanol, dried at 80 °C and then was reused for the next cycle of catalysis. As shown in Fig. 11, the catalyst was recycled several times with a little loss in catalytic performance after the third cycle. This can be explained by the reduction of silver (Ag^+^ → Ag^0^) by the excited electrons during catalytic processes, confirmed by the gradual colour change of the Ag_3_PO_4_ catalyst (From yellow to dark brown), resulting in decrease in the catalytic efficiency.

### Antibacterial activity studies

**Fig. 12:**
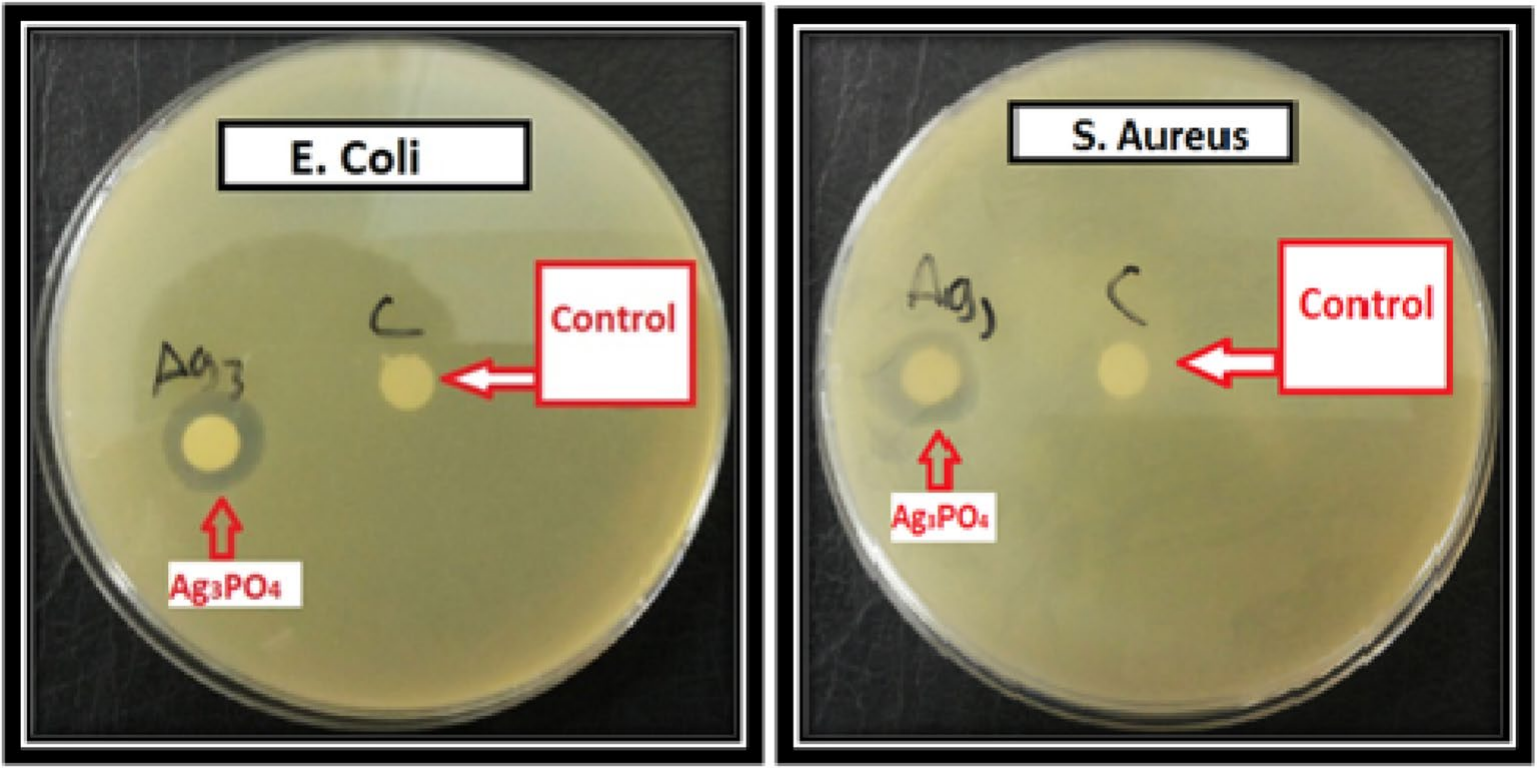
Antibacterial test results for E. *Coli* and S. *Aureus* after 24 h of incubation using nanostructured Ag_3_PO_4_ (1 mg/mL) and water as negative control

The production of a large quantity of Ag_3_PO_4_ through a simple and economical method from natural phosphate can be employed as an antibacterial agent suitable for the biological treatment of wastewater. In this optic, the obtained results of antibacterial activity of Ag_3_PO_4_ against E. *Coli* (Gram-negative) and S. *Aureus* (Gram-positive) are shown in Fig. 12. The zone of inhibition clearly indicated the significant antibacterial effect of the nanostructured Ag_3_PO_4_ as quantitatively shown in Table 2. With 1 mg/mL as the serial concentration of Ag_3_PO_4_ in the biological solution, the maximum diameters of the zones of inhibition are approximately 12.01 mm and 13.50 mm against S. aureus and E. coli, respectively. As a result, the nanostructured powder of Ag_3_PO_4_ prepared from phosphate rock is in fact an effective antibacterial agent on Gram positive and Gram-negative bacteria such as largely described in the literature.

**Table 2:** The inhibition diameter zone of the nanostructured Ag_3_PO_4_ against *E. Coli* and *S. Aureus* at different concentrations

Concentration (mg/mL)	Zone of inhibition (mm)	
	E. *coli*	S. *Aureus*
1	13.50	12.01
0.5	12.54	11.33
0.25	10.20	10.9
0.125	10.00	9.2

**Fig. 13:**
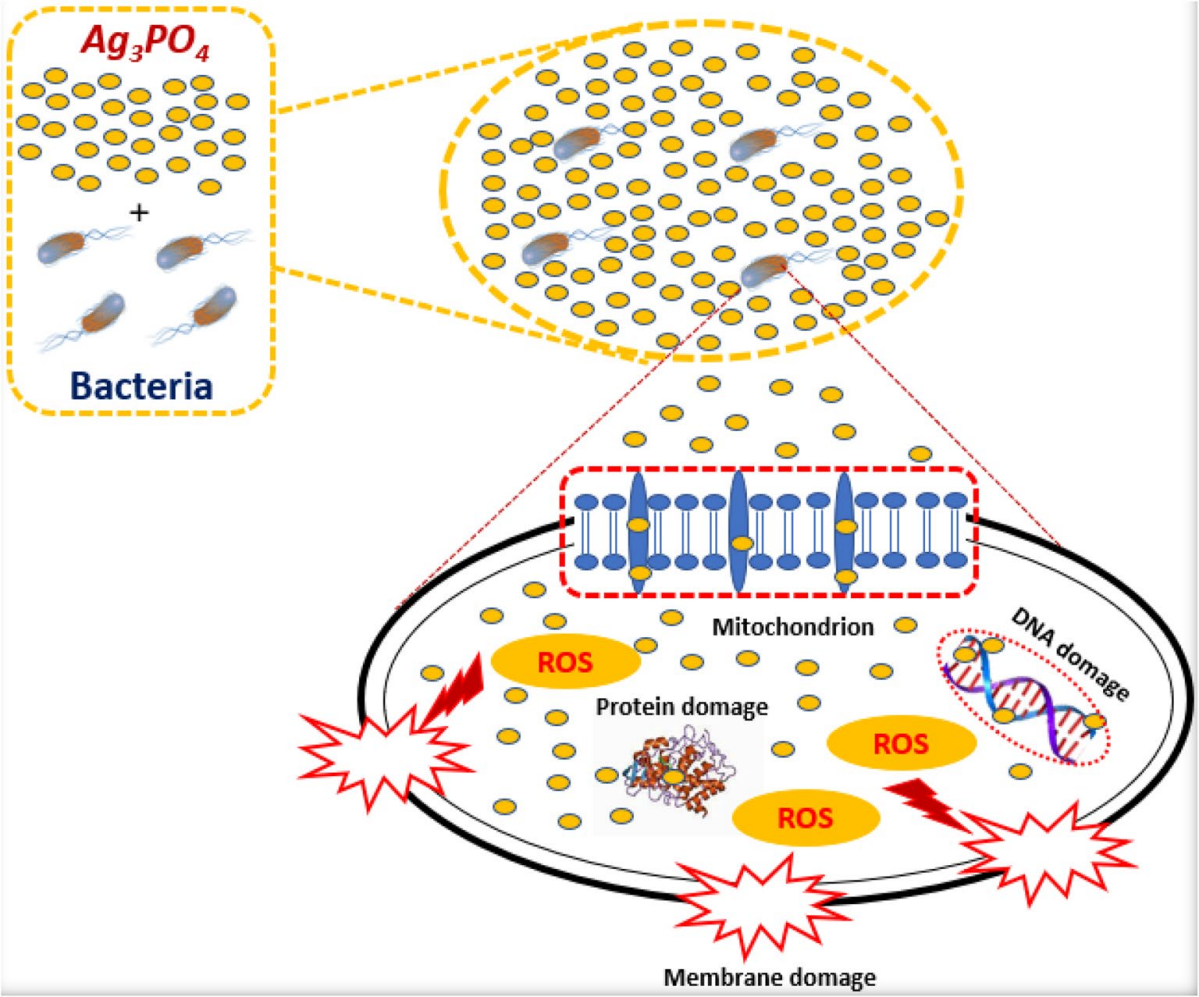
Possible antibacterial mechanism of action of Ag_3_PO_4_ prepared from natural phosphate rock

According to some research reports [38–41], the plausible antibacterial mechanism of the nanostructured Ag_3_PO_4_ prepared from natural phosphate was proposed as follows (Fig. 13): (i) Initially, due to electrostatic attraction and affinity to sulfur proteins, our proper material can adhere to the cell wall and cytoplasmic membrane. (ii) The adhered material can enhance the permeability of the cytoplasmic membrane and lead to disruption of the bacterial envelope. (iii) After the destruction of membrane system, a large number of Ag_3_PO_4_, can enter into the cells. (iv) The final stage involves the interference via Ag_3_PO_4_ directly binding with proteins, lipids, enzymes, DNA and the oxidation of them by generating reactive oxygen species (ROS). As Known, the generation of ROS and oxidative stress are more significant mechanism owing to the strong destructive power. Subsequently, the integrity of the cell wall/membrane was disrupted, and the intracellular contents leaked out which consequently results in cell death.

## Conclusion

This study develops a novel approach for the synthesis of the single phase of the nanostructured Ag_3_PO_4_ from natural phosphate as phosphate source via dissolution–precipitation process. The as-prepared sample was successfully characterized by various physicochemical techniques in order to study its structural, textural and morphological properties. Then, the nanostructured catalyst exhibited higher catalytic activity towards the reduction of 4-nitrophenol to 4-aminophenol using NaBH_4_ as reducing agent in aqueous solution. In addition, the prepared Ag_3_PO_4_ catalyst possesses significant antibacterial activities against E. *Coli* and S. *Aureus* bacteria. The operational simplicity, short reaction times, recyclability and antibacterial activity are the outstanding features of the present study.

## Data Availability

All data generated or analysed during this study are included in this published article.

## Abbreviations

FTIR: Fourrier Transform infrared
XRD: X-ray diffraction
SEM: Scanning electron microscope
TEM: Transmission electron microscope
IUPAC: International Union of Pure and Applied Chemistry
EDS: Energy dispersive spectroscopy
STEM: The scanning-transmission electron microscope
^31^PMAS-NMR: Magic angle spinning in solid-state nuclear magnetic resonance spectroscopy
ROS: Reactive oxygen species

## References

[1] Hoffmann MR, Martin ST, Choi W, Bahnemann DW (1995). Environmental applicationsof semiconductor photocatalysis. Chem Rev.

[2] Chen C, Ma W, Zhao J (2010). Semiconductor-mediated Photodegradation of pollutants under visible-light irradiation. ChemSoc Rev.

[3] Higson FK (1992). Microbial degradation of nitroaromatic compounds. Adv Appl Microbiol.

[4] Rodriguez I, Liompart MP, Cela R (2000). Solid-phase extraction of phenols. J Chromatogr.

[5] Feng J, Su L, Ma Y, Ren C, Guo Q, Chen X (2013). CuFe_2_O_4_ magnetic nanoparticles: a simple and efficient catalyst for the reduction of nitrophenol. ChemEng J.

[6] Komatsu T, Hirose T (2004). Gas phase synthesis of para-aminophenol from nitrobenzene on Pt/zeolite catalysts. Appl Catal A.

[7] Rode CV, Vaidya MJ, Jaganathan R, Chaudhari RV (2001). Hydrogenation of nitrobenzene top-aminophenol in a four-phase reactor: reaction kinetics and mass transfer effects. Chem Eng Sci.

[8] Vaidya MJ, Kulkami SM, Chaudhari RV (2003). Synthesis of p-Aminophenol by CatalyticHydrogenation of p-nitrophenol. Org Process Res Dev.

[9] Liang Q, Ma W, Shi Y, Li Z, Yang X (2012). Hierarchical Ag_3_PO_4_ porous microcubes with enhanced photocatalytic properties synthesized with the assistance of trisodium citrate. Cryst EngComm..

[10] Khan A, Qamar M, Muneer M (2012). Synthesis of highly active visible-light-driven colloidal silver orthophosphate. ChemPhysLett.

[11] Bi Y, Hu H, Ouyang S, Lu G, Caob J, Ye J (2012). Photocatalytic and photoelectric properties of cubic Ag_3_PO_4_ sub-microcrystals with sharp corners and edges. Chem Commun.

[12] Wang WG, Cheng B, Yu JG, Liu G, Fan WH (2012). Visible light photocatalytic activity and deactivation mechanism of Ag_3_PO_4_ spherical particles. Chem Asian J.

[13] Yahui Z, Xiaochen Z, Ruiming H, Yang Y, Ping L, Qingsheng W (2019). Bifunctional nano-Ag_3_PO_4_ with capabilities of enhancing ceftazidime for sterilization and removing residues. RSC Adv.

[14] Yaling L, Hangyu Zh, Genxing Zh, Changyu S, Haihua P, Xurong X, Ruikang T (2015). High efficient multifunctional Ag_3_PO_4_ loaded hydroxyapatite nanowires for water treatment. J of Hazard Mater.

[15] Sulaeman U, Wu X, Liu B, Yin S, Sato T (2015). Synthesis of Ag_3_PO_4_-polyvinyl alcohol hybrid microcrystal with enhanced visible light photocatalytic activity. Appl Surf Sci.

[16] Li X, Zheng R, Luo Q, Wang D, An J, Yin R, Liu Y, Wu D, Han X (2015). Cyclized polyacrynitrile modified Ag_3_PO_4_ photocatalysts with enhanced photocatalytic activity under visible-light irradiation. Appl Surf Sci.

[17] Lin X, Guo X, Shi W, Guo F, Che G, Zhai H, Yan Y, Wang Q (2015). Ag_3_PO_4_ quantum dots loaded on the surface of leaf-like InVO_4_/BiVO_4_ heterojunction with enhanced photocatalytic activity. Catal Commun.

[18] Mehraj O, Mir AN, Pirzada BM, Sabir S (2015). Fabrication of novel Ag_3_PO_4_/BiOBr heterojunction with high stability and enhanced visible-light-driven photocatalytic activity. Appl Surf Sci.

[19] Zahouily M, Salah M, Bahlaouane B, Rayadh A, Houmam A, Hamed Sebti S (2004). Solid catalysts for the production of fine chemicals: the use of natural phosphate alone and doped base catalysts for the synthesis of unsaturated arylsulfones. Tetrahedron.

[20] Zahouily M, Mezdar A, Rakik J, Elmakssoudi A, Rayadh A, Sebti S (2005). A mild and efficient method for the protection of carbonyl compounds as dithioacetals, dithiolanes and dithianes catalysed by iodine supported on natural phosphate. J Mol Catal A Chem.

[21] Zahouily M, Elmakssoudi A, Mezdar A, Rayadh A, Sebti S (2007). Natural phosphate and potassium fluoride doped natural phosphate catalysed simple one-pot synthesis of α-amino phosphonates under solvent-free conditions at room temperature. CatalCommun.

[22] Dânoun K, Jioui I, Bouhrara M, Zahouily M, Solhy A, Jouiad M, Len C, Fihri A (2015). Nanostructured pyrophosphate Na_2_CaP_2_O_7_ as catalyst for selective synthesis of 1,2-disubstituted benzimidazoles in pure water. Curr OrgChem.

[23] Jioui I, Dânoun K, Solhy A, Jouiad M, Zahouily M, Essaid B, Len C, Fihri A (2016). Modified fluorapatite as highly efficient catalyst for the synthesis of chalcones via Claisen-Schmidt condensation reaction. J Ind Eng Chem.

[24] Dânoun K, Essamlali Y, Amadine O, Tabit R, Fihri A, Len C, Zahouily M (2018). Nanostructured pyrophosphate Na_2_PdP_2_O_7_-catalyzed suzuki-miyaura cross-coupling under microwave irradiation. Appl Organomet Chem.

[25] Tabit R, Amadine O, Essamlali Y, Dânoun K, Rhihil A, Zahouily M (2018). Magnetic CoFe_2_O_4_nanoparticles supported on graphene oxide (CoFe_2_O_4_/GO) with high catalytic activity for peroxymonosulfate activation and degradation of rhodamine B. RSC Adv.

[26] Amedlous A, Amadine O, Essamlali Y, Dânoun K, Aadil M, Zahouily M (2019). Aqueous-phase catalytic hydroxylation of phenol with H_2_O_2_by using a copper incorporated apatite nanocatalyst. RSC Adv.

[27] Dânoun K, Essamlali Y, Amadine O, Mahi H, Zahouily M (2020). Eco-friendly approach to access of quinoxaline derivatives using nanostructured pyrophosphate Na_2_PdP_2_O_7_as a new, efficient and reusable heterogeneous catalyst. BMC Chemistry.

[28] Zhang R, Zhang T, Cai Y, Zhu X, Han Q, Li Y, Liu Y (2020). Reduced graphene oxide-doped Ag_3_PO_4_ nanostructure as a high efficiency photocatalyst under visible light. J Inorg Organomet Polym Mater.

[29] Machida N, Kawachi M, Uedaa A, Shigematsu T, Takahashi M (1995). Mixed anion effect of silver ion conducting glasses in the systems AgI-Ag_2_MoO_4_-Ag_3_PO_4_ and AgI-Ag_2_MoO_4_-Ag_2_PO_3.5_ and structural study by ^31^P MAS-NMR. Solid State Ionics.

[30] Jana S, Ghosh SK, Nath S, Pande S, Praharaj S, Panigrahi S, Basu S, Endo T, Pal T (2006). Synthesis of silver nanoshell-coated cationic polystyrene beads: a solid phase catalyst for the reduction of 4-nitrophenol. Appl Catal.

[31] Ghosh SK, Mandal M, Kundu S, Nath S, Pa T (2004). Bimetallic Pt–Ni nanoparticles can catalyze reduction of aromatic nitro compounds by sodium borohydride in aqueous solution. Appl Catal A Gen.

[32] Deng Y, Cai Y, Sun Z, Liu J, Liu C, Wei J, Li W, Wang Y, Zhao D (2010). Multifunctional mesoporous composite microspheres with well-designed nanostructure: a highly integrated catalyst system. J Am ChemSoc.

[33] Zhang Y, Gao G, Qian Q, Cui D (2012). Chloroplasts-mediated biosynthesis of nanoscale Au-Ag alloy for 2-butanone assay based on electrochemical sensor. Nanoscale Res Lett.

[34] Koga H, Umemura Y, Kitaoka T (2011). In situ synthesis of bimetallic hybrid nanocatalysts on a paper-structured matrix for catalytic applications. Catalysis.

[35] Layek K, Kantam ML, Shirai M, Ni-Hamane D, Sasaki T, Maheswaran H (2012). Gold nanoparticles stabilized on nanocrystalline magnesium oxide as an active catalyst for reduction of nitroarenes in aqueous medium at room temperature. Green Chem.

[36] Zhang P, Shao C, Zhang Z, Zhang M, Mu J, Guoab Z, Liua Y (2011). In situ assembly of well-dispersed Ag nanoparticles (AgNPs) on electrospun carbon nanofibers (CNFs) for catalytic reduction of 4-nitrophenol. Nanoscale.

[37] Bendi R, Imae T (2013). Renewable catalyst with Cu nanoparticles embedded into cellulose nano-fiber film. RSC Adv.

[38] Gomaa EZ (2017). Silver nanoparticles as an antimicrobial agent: a case study on Staphylococcus aureus and Escherichia coli as models for Gram-positive and Gram-negative bacteria. J Gen Appl Microbiol.

[39] Das B, Dash S, Mandal D, Adhikary J, Chattopadhyay S, Tripathy S, Dey A, Manna S, Dey S, Das D, Roy S (2016). BLDE Univ. Green-synthesized silver nanoparticles kill virulent multidrug-resistant *Pseudomonas aeruginosa* strains: a mechanistic study. J Health Sci.

[40] Krishnaraj C, Jagan EG, Rajasekar S, Selvakumar P, Kalaichelvan PT, Mohan N (2010). Synthesis of silver nanoparticles using *Acalypha indica* leaf extracts and its antibacterial activity against water borne pathogens. Colloids Surf B.

[41] Abalkhil TA, Alharbi SA, Salmen SH, Wainwright M (2017). Bactericidal activity of biosynthesized silver nanoparticles against human pathogenic bacteria. Biotechnol Biotechnol Equip.

